# Bone marrow mesenchymal stem cells-derived exosomal miR-145-5p reduced non-small cell lung cancer cell progression by targeting SOX9

**DOI:** 10.1186/s12885-024-12523-z

**Published:** 2024-07-22

**Authors:** Wu Yan, Haiyu Yang, Dekun Duan, Yufeng Wu, Youhu Liu, Jianping Mao, Yong Zhao, Junsong Ye

**Affiliations:** 1 Jiangxi Beizheng Stem Cell Science Co. Ltd., Ganzhou, Jiangxi, 341000 PR China; 2 Subcenter for Stem Cell Clinical Translation, First Affiliated Hospital of Gannan Medical University, Ganzhou, Jiangxi, 341000 PR China; 3 Drugs and Medical Devices Clinical Trial Center, First Affiliated Hospital of Gannan Medical University, Ganzhou, Jiangxi 341000 PR China

**Keywords:** NSCLC, Exosomes, miR-145-5p, SOX9

## Abstract

**Background:**

The role of miR-145-5p in non-small cell lung cancer (NSCLC) has been studied, however, the regulation of hBMSCs-derived exosomes (Exo) transmitted miR-145-5p in NSCLC was still unknown. This study aimed to investigate the role of hBMSCs-derived exosomes (Exo) in the progression of NSCLC.

**Methods:**

The Exo was extracted from hBMSCs and added to A549 and H1299 cell culture, followed by the detection of cell proliferation, migration, and invasion. The correlation between the expression of miR-145-5p and SOX9, as well as their binding relationship was determined by correlation analysis, luciferase gene reporter assay and RNA pull-down assays. The in vivo animal model was established to further verify the impact of hBMSCs-Exo.

**Results:**

It showed that miR-145-5p was downregulated and SOX9 was upregulated in NSCLC tissues. HBMSCs-derived Exo, and hBMSCs-Exo with overexpression of miR-145-5p could inhibit cell proliferation, migration, and invasion of both A549 and H1299 cells, and prevent against tumor progression in vivo. MiR-145-5p and SOX9 were found to be able to bind to each other, and a negative correlation were observed between the expression of them in NSCLC tissues. Furthermore, inhibition of SOX9 could reversed the suppressed role of miR-145-5p in vitro and in vivo.

**Conclusion:**

Therefore, HBMSCs-Exo effectively transmitted miR-145-5p, leading to the suppression of malignant development in NSCLC through the regulation of SOX9.

**Supplementary Information:**

The online version contains supplementary material available at 10.1186/s12885-024-12523-z.

## Introduction

Lung cancer is becoming a common malignancy in the world, and causes high incidence and mortality [1, 2]. As a major subtype of lung cancer, non-small cell lung cancer (NSCLC) represents approximately 85% of all lung cancer cases [3]. Despite advancements in diagnostic techniques and therapies, the prognosis of patients with this disease remains unsatisfactory. Therefore, there is an urgent need to develop novel therapeutic strategies to improve outcomes for these patients.

Stem cells derived exosomes (Exos) are membrane-derived nano-vesicles that play essential roles in various biological processes [4]. It has been reported that exosomal microRNAs (miRNAs) derived from stem cells could impact the pathogenetic processes in various diseases. For example, human umbilical cord mesenchymal stem cells-derived Exos promote the cell growth of pancreatic ductal adenocarcinoma cells via transferring miR-100-5p [5]. HBMSC-secreted Exos containing miR-361-5p can efficiently alleviate chondrocyte damage, and then alleviates osteoarthritis [6]. These findings suggested that stem cells-derived Exos might be a promising strategy for disease treatment. Recently, it is reported that miR-145-5p was closely involved in the progression of NSCLC. MiR-145-5p inhibits the process of epithelial-mesenchymal transition (EMT) in NSCLC cells [7]. MiR-145-5p prevents the development of NSCLC via the regulation of lncRNA LINC00852 and KLF4 [8]. One previous study reported that human bone marrow mesenchymal stem cells (hBMSCs)-derived miR-145-5p containing Exos could reduce inflammation level, and then attenuate spinal cord injury [9]. Therefore, we hypothesized that exosomes derived from hBMSCs containing miR-145-5p might have an impact on the development of NSCLC. This study was performed to test this hypothesis.

SOX9, a member of the SOX (sex-determining region Y-related) family of transcriptional regulators, is typically silenced in normal tissues but is significantly upregulated in various cancers, including NSCLC [10–12]. Previous studies confirmed the binding relationship between miR-145-5p and SOX9 in different types of cell contexts such as chondrogenic differentiation [13], gastric cancer [14], and glioma [15]. In the present study, we revealed that hBMSCs-derived Exos could inhibit the NSCLC progression via exosomal miR-145-5p. Meanwhile, we also found the binding interaction and negative correlation between the expression of miR-145-5p and SOX9. In comparison to previous studies which explored the relationship between miR-145-5p and SOX9, our findings provided novel insights into the regulatory role of this axis in NSCLC. Specifically, our study demonstrates that hBMSCs-miR-145-5p containing Exos can effectively prevent the progression of NSCLC by regulating SOX9.

## Materials and methods

### Clinical samples

A total of 30 pairs of NSCLC tumor tissues and adjacent normal tissues were collected from NSCLC patients at the First Affiliated Hospital of Gannan Medical University between 2016 to 2020. All patients have signed the informed consent. This study was approved by the First Affiliated Hospital of Gannan Medical University (No. 87655). Animal experiments was in accordance with ARRIVE guidelines. All tissue samples were stored in -80 °C before use. Ethical approval numbers: No.43922.

### Cell culture

Human bone marrow mesenchymal stem cells (BMSCs), and NSCLC cell lines A549 and H1299 were purchased from American Type Culture Collection (MD, USA). Cells were grown in DMEM/F12 medium containing 10% FBS (Gibco, USA), 100 U/mL penicillin, and 100 μg/mL streptomycin (Gibco) in a 37 °C humid environment with 5% CO_2_.

### Characterization of hBMSCs and exosomes

The hBMSCs at passages 4–6 were cultured overnight, and the cellular morphology was examined under an inverted phase contrast microscope. The hBMSCs-derived exosomes (Exo) were isolated via ultra-high-speed centrifugation as previously reported [16, 17]. In brief, hBMSCs at passages 4–6 were cultured in 6-well plates with a cell density of 5 × 10^5^ cells per well, totaling 12 ml of serum-free medium. After that, the supernatant was harvested when changing fresh medium. The supernatant was centrifuged at 12,000 r/min for 40 min to remove cell residues. Then exosome extraction reagent was added into the supernatant and incubated at 4 °C overnight. Finally, ultra-high-speed centrifugation was performed, and the precipitate was re-suspended in PBS as the exosomes and stored at − 80 °C. The concentration of exosomes were measured by BCA method. The morphology of exosomes was examined by a transmission electron microscope, and the exosomes-related surface markers (Tsg101, CD63 and Calnexin) were detected by Western blot analysis.

### Transfection

The 100 nM miR-145-5p mimics and inhibitors, along with their respective control miR-NC and inhibitor NC, and si-ROX9, si-NC were obtained from Shanghai GenePharma Co. Ltd. These molecules were then transfected into A549, and H1299 cells using Lipofectamine 2000 reagent (Invitrogen).

### Preparation of Exo-miR-145-5p mimics and other Exos

First, 5 × 10^6^ hBMSCs per dish were seeded into 10 cm dishes (total 6 dishes). The next day, miR-NC, miR-145-5p mimics, inhibitor NC, miR-145-5p inhibitor, miR-145-5p inhibitor + si-SOX9 and miR-145-5p inhibitor + si-NC were transfected into hBMSCs using 60 µL Lipofectamine 2000 reagent (Invitrogen). After incubating these cells for 48 h, the medium was changed with serum-free medium for 24 h. Next, we collected the supernatant and extracted exosome following the steps mentioned above. The relative miR-145-5p and SOX9 mRNA expression were calculated by qRT-PCR to verify these molecules were loaded.

### Exosome treatment

To investigate its role, cells were transfected with hBMSCs-Exo carrying different molecules, and labeled as Exo-miR-NC, Exo-miR-145-5p mimics, Exo-inhibitor NC, Exo-miR-145-5p inhibitor, Exo-miR-145-5p inhibitor + si-SOX9, and Exo-miR-145-5p inhibitor + si-NC. Subsequently, 50 μg of hBMSCs-derived exosomes with these molecules were added to the cell cultures of A549 or H1299, and incubated for two days. After this incubation period, the cells were utilized for other functional assays.

### Cell proliferation

Cell viabilities were assessed using the CCK8 kit (Dojindo, Rockville, MD, USA) as previously reported [18]. The optical density at 450 nm at different time points were detected using a spectrophotometer. For EdU staining, the BeyoClick EdU Cell Proliferation Kit (Beyotime Biotechnology, Shanghai, China) was used. Briefly, the transfected cells were incubated with 10 μM Edu for 2 h, followed by the incubation of DAPI solution for nuclei. Staining images were observed under a fluorescence microscope. EdU positive cells (proliferating cells) % = EdU positive cells/total cells × 100% from three random visual fields.

### Transwell assay

An 8-μm well size Transwell chamber with well inserts (Corning, N.Y., USA) was used for invasion assay. To promote cell invasion, 100 μL of 50 mg/L Matrigel (1:40 dilution) was coated onto the upper surface of chamber’s bottom membrane. A cell suspension containing 2 × 10^5^ cells in 100 μL was added to the upper chamber, while the lower chamber was filled with 600 µL of DMEM medium containing 20% FBS. After 24 h, cells were fixed by methanol for 10 min and stained with 0.1% crystal violet for imaging and counting under five random fields using optical microscope with 400 × magnification. The five random fields were obtained from the left, right, up, down and center of the chamber. The cell number was showed by mean ± standard deviation and compared by Student's *t* test. Cell migration assay was performed the same as invasion assay without coating the Matrigel.

### Apoptosis analysis

A total of 1 × 10^5^ cells were seeded into 6-well plates and cultured overnight, and the supernatant was removed by centrifugation. According to the Annexin VFITC cell apoptosis detection kit (Biovision, USA), cell pellets were incubated with 500 μL loading buffer, 5 μL Annexin V-FITC and 10 μL propidium iodide (PI) solution for 20 min. Cell apoptosis was evaluated with the flow cytometry (BD Biosciences). Apoptosis was quantified following Annexin-V/PI staining % were only quadrants Q2/Q3 combined.

### qRT-PCR

Total RNAs were isolated using TRIzol reagent (Invitrogen). The cDNA was synthesized using the specific Reverse Transcription Kit (Applied Biosystems). PCR reactions were performed using SYBR green Super-mix (Thermo Fisher) on an ABI 7500 PCR detection system. Gene expression was quantified the 2^–ΔΔCT^ method. GAPDH/U6 was used as the normalization control. Primer sequences were: miR-145-5p forward 5′-ACACTCCAGCTGGGTCCCTAAGGACCCTTTT-3′ and reverse 5- CTCAACTGGTGTCGTGGAGTCGGCAATTCAGTTGAGCAGGTCAA-3′; and SOX9 forward 5′- AGCGAACGCACATCAAGAC-3′ and reverse 5′-CTGTAGGCGATCTGTTGGGG-3′. U6 F: 5′-CTCGCTTCGGCAGCACA-3′, R: 5′-AACGCTTCACGAATTTGCGT-3′; GAPDH F: 5′-TCCCATCACCATCTTCCA-3′, R: 5′-CATCACGCCACAGTTTTCC-3′.

### Western blot analysis

Total proteins were extracted using RIPA buffer. Approximately 50 µg of protein sample per lane was separated by 12% SDS-PAGE and subsequently transferred onto PVDF membranes, followed by incubation with the appropriate primary antibodies CD63 (1:1,000) (ab134045, abcam), Tsg101 (1:1,000) (ab125011, abcam), Calnexin (1:1,000) (ab227310, abcam), and SOX9 (1:1,000) (ab185966, abcam) at 4 °C for overnight. The next day, the membrane was subjected to HRP-conjugated secondary antibody (1:10,000) for 1 h. All antibodies were purchased from Abcam. The protein bands were visualized using a chemiluminescence reagent, and data analysis was conducted using Image J software.

### Luciferase reporter assay

The wild type and mutant sequences of the SOX9 3′-UTR, containing binding sites of miR-145-5p, were cloned downstream of the pmirGLO reporter vector (that it is a SOX9 3'UTR luciferase reporter vector). The H1299 cells were co-transfected with luciferase reporter vector, miR mimic or miR-NC for two days. The relative luciferase activity was measured using a Dual-Luciferase reporter assay system and subsequently normalized to *Renilla* activity.

### RNA pull-down assay

The RNA of patient samples were extracted using MolPure® Cell/Tissue Total RNA Kit (19221ES50, YESEN). Biotin labelled and biotin non-labelled SOX9 probes (Bio-SOX9 and Bio-NC) were synthesize by Shanghai GenePharma Co., Ltd. H1299 cells were lysed and the whole lysates were incubated with probe-coated M-280 streptavidin-magnetic beads (Invitrogen) at 4 °C overnight. Then RNAs were extracted, and the expression of miR-145-5p was detected using qRT-PCR analysis.

### Tumor xenograft in vivo model

A total of 25 male BALB/c nude mice which lack mature T cells through the thymus (6 weeks old) were obtained from Beijing Vital River Laboratory Animal Technology Co., Ltd. were used to establish the in vivo model. Each mouse was subcutaneously injected with 1 × 10^6^ A549 cells. Once the mean tumor volume reached 100 mm^3^, all mice were randomly divided into five groups (each group consisted of 5 mice): Control group (PBS injection), Exo-miR inhibitor-NC group, Exo-miR inhibtor group, Exo miR-145-5p inhibitor + si-NC group, and Exo miR-145-5p inhibitor + si-SOX9 group. Approximately 200 μg/mouse hBMSCs-derived Exo was intratumorally injected into mice every two days for a total of 10 times. Four weeks later, when the longest tumour diameter reached 1.5 cm, the mice were euthanized using asphyxiation in a CO_2_ chamber, and tumors were weighted. Tumor volume was determined using the formula (length × width^2^)/2. This study was approved by First Affiliated Hospital of Gannan Medical University (No. 76395).

### Statistical analysis

Data were presented as the mean ± standard deviation (SD), and each experiment was repeated for three independent times. Student's *t* test was used to conduct comparison between two groups. One-way-ANOVA followed by Tukey’s multiple comparisons test was applied for multi-group comparison. Statistical significance was set at *p* < 0.05.

## Results

### Characterization of hBMSCs-derived exosomes

**Fig. 1:**
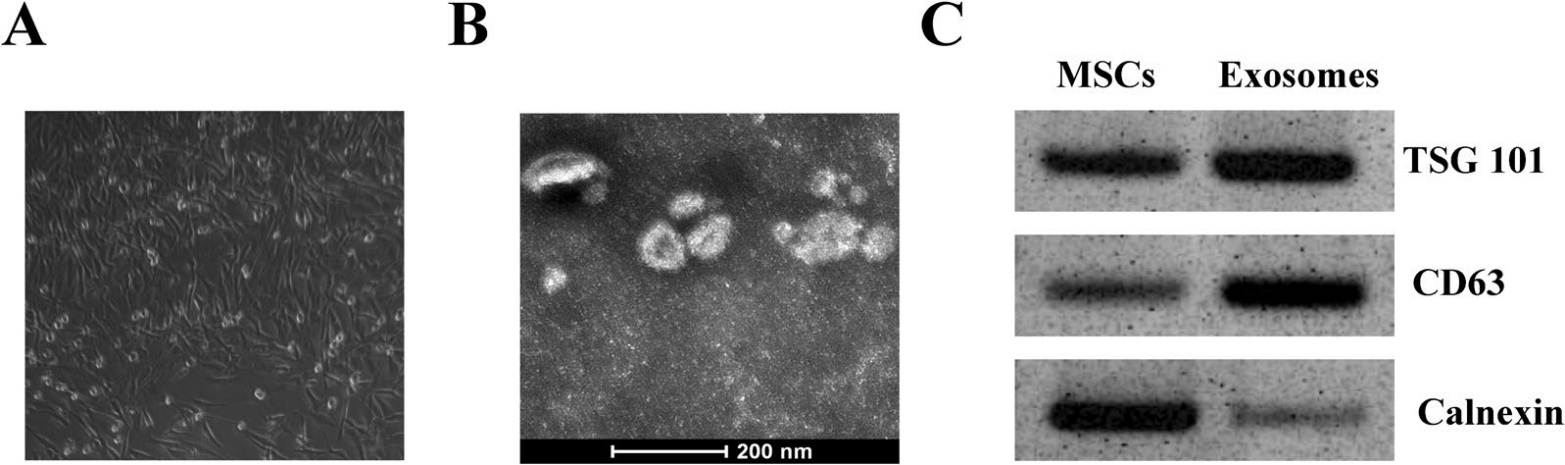
Characterization of hBMSCs-derived exosomes. **A** The morphology of hBMSCs by inverted microscope. magnification × 400. **B** The morphology of hBMSCs-Exo by a TEM. scale bar = 200 nm. **C** Western blot analysis of Exo-surface markers in hBMSCs-Exo and hBMSCs

Prior to exosome isolation, we observed the hBMSCs under an inverted microscope. Fig. 1A illustrated that the cells exhibited various morphologies, including large, polygonal, spindle-like, or spindle-shaped adherent cells, with prominent nuclei located centrally within the hBMSCs. Then exosomes were extracted, and we found that exosomes had characteristic morphological characteristics and their diameter is approximately from 40 to 100 nm (Fig. 1B). In addition, Western blot analysis showed that the expression levels of exosomes-related surface markers Tsg101 and CD63 were increased, while the expression levels of Calnexin were reduced in hBMSCs-Exo than that in Exo-removing hBMSCs (Fig. 1C). These results suggested that the isolated exosomes are suitable for our study.

### HBMSCs-Exo reduced cell malignant phenotype in vitro

**Fig. 2:**
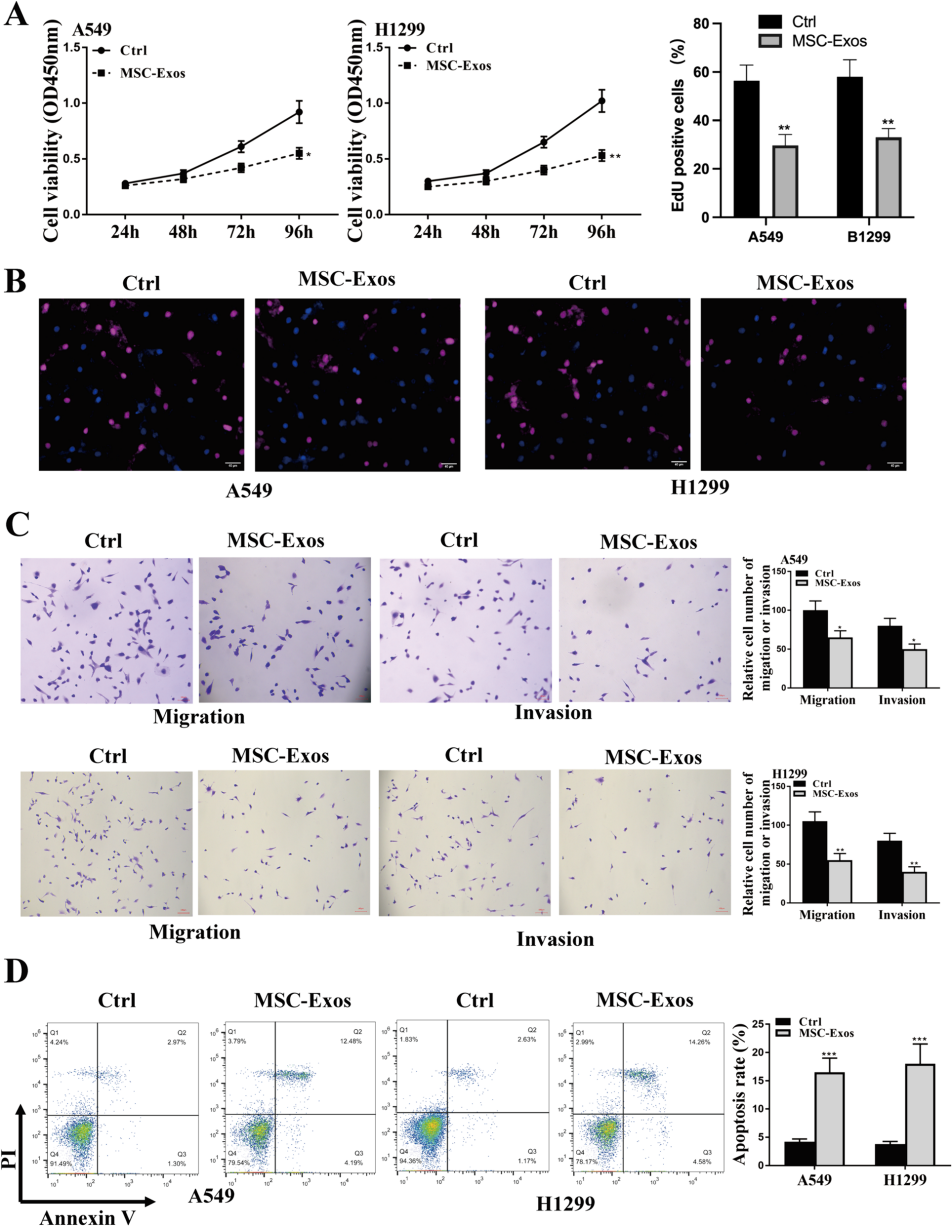
The impacts of hBMSCs-Exo in NSCLC cell progression. 200 μg hBMSCs-derived exosomes were added into cell culture of A549 and H1299, and stimulated for two days. **A** CCK-8 assay. **B** EdU staining assay. scale bar = 40 μm. **C** Transwell assay for cell invasion and migration. scale bar = 100 μm. **D** Flow cytometry analysis for apoptosis. * *p* < 0.05, ** *p* < 0.01, *** *p* < 0.001

To investigate the impact of hBMSCs-Exo, the Exo of original hBMSCs was extracted and used to treat A549 and H1299 cells. As shown in Fig. 2A-D, the cell proliferation, migration, and invasion of the two cell lines were significantly inhibited, and the apoptotic rate of cells was enhanced by hBMSCs-Exo in comparison to PBS control. These results suggested that hBMSCs-Exo could efficiently reduce NSCLC progression in vitro.

### HBMSCs-Exo overexpressing miR-145-p demonstrated a reduction in cell malignant phenotype in vitro

**Fig. 3:**
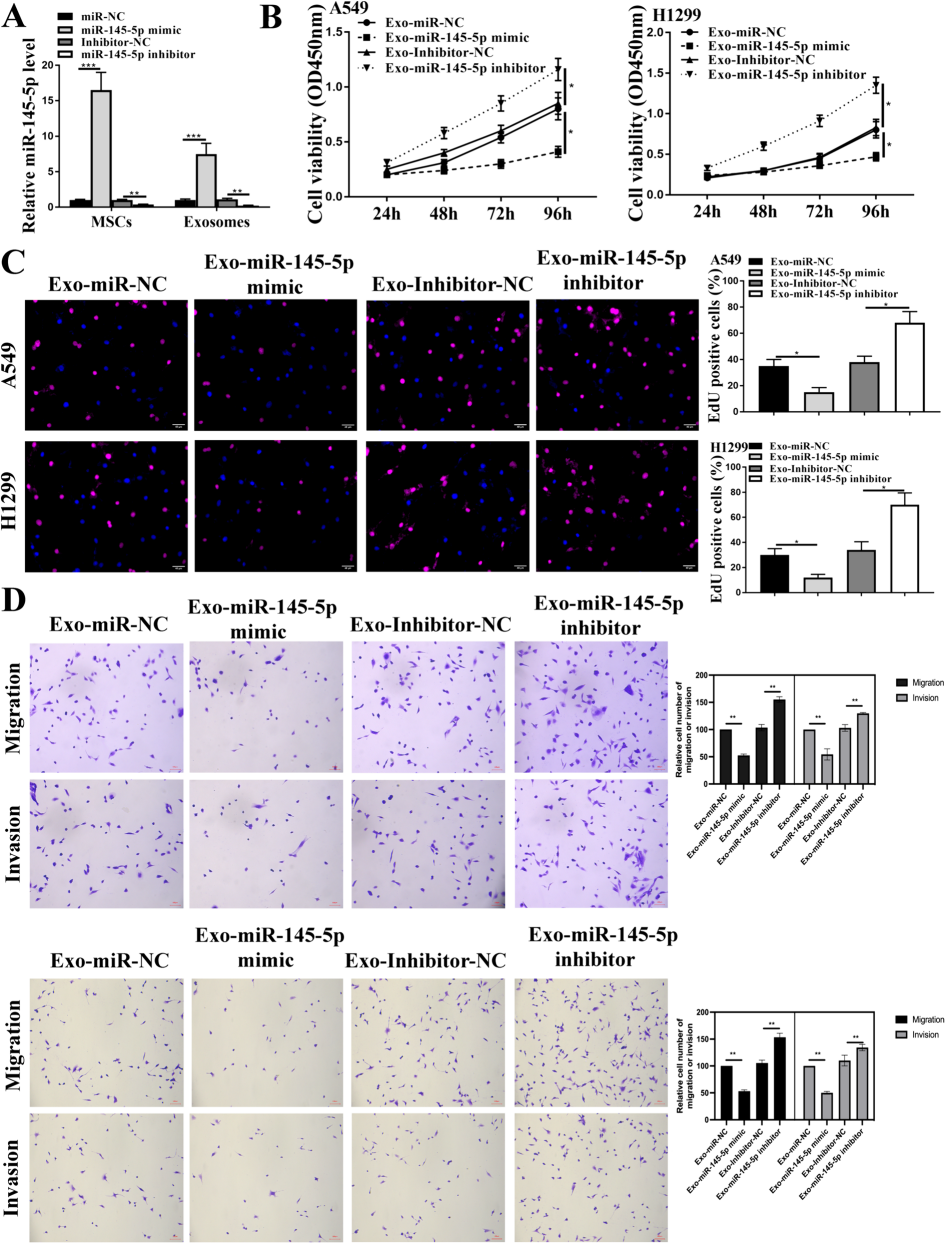
The impact of hBMSCs-Exo with overexpressed or reduced miR-145-5p level in NSCLC cell progression. MiR-145-5p mimics/inhibitor were introduced into hBMSCs, and exosomes were extracted to stimulate A549 and H1299 cells for two days. **A** qRT-PCR detection of miR-145-5p in hBMSCs and hBMSCs-Exo. **B** CCK-8 assay. **C** EdU staining assay. scale bar = 40 μm. **D** Transwell assay for cell invasion and migration. scale bar = 100 μm. * *p* < 0.05, ** *p* < 0.01, *** *p* < 0.001

To further elucidate the role of miR-145-5p in NSCLC cell function, we transfected miR mimics and inhibitors into hBMSCs and extracted Exo. Our analysis revealed that miR-145-5p mimics significantly increased its expression, whereas miR inhibitor reduced its level both in hBMSCs and hBMSCs-Exo (Fig. 3A). Subsequently, through a series of functional assays, we demonstrated that hBMSCs-Exo carrying miR-145-5p mimics significantly suppressed cell proliferation, migration, and invasion in both cell types. On the other hand, hBMSCs-Exo with miR-145-5p inhibitor promoted a more malignant phenotype in these cells (Fig. 3B-D). These results strongly suggest that the impact of hBMSCs-Exo on NSCLC cells is mediated by miR-145-5p.

### SOX9 was a downstream target gene of miR-145-5p

**Fig. 4:**
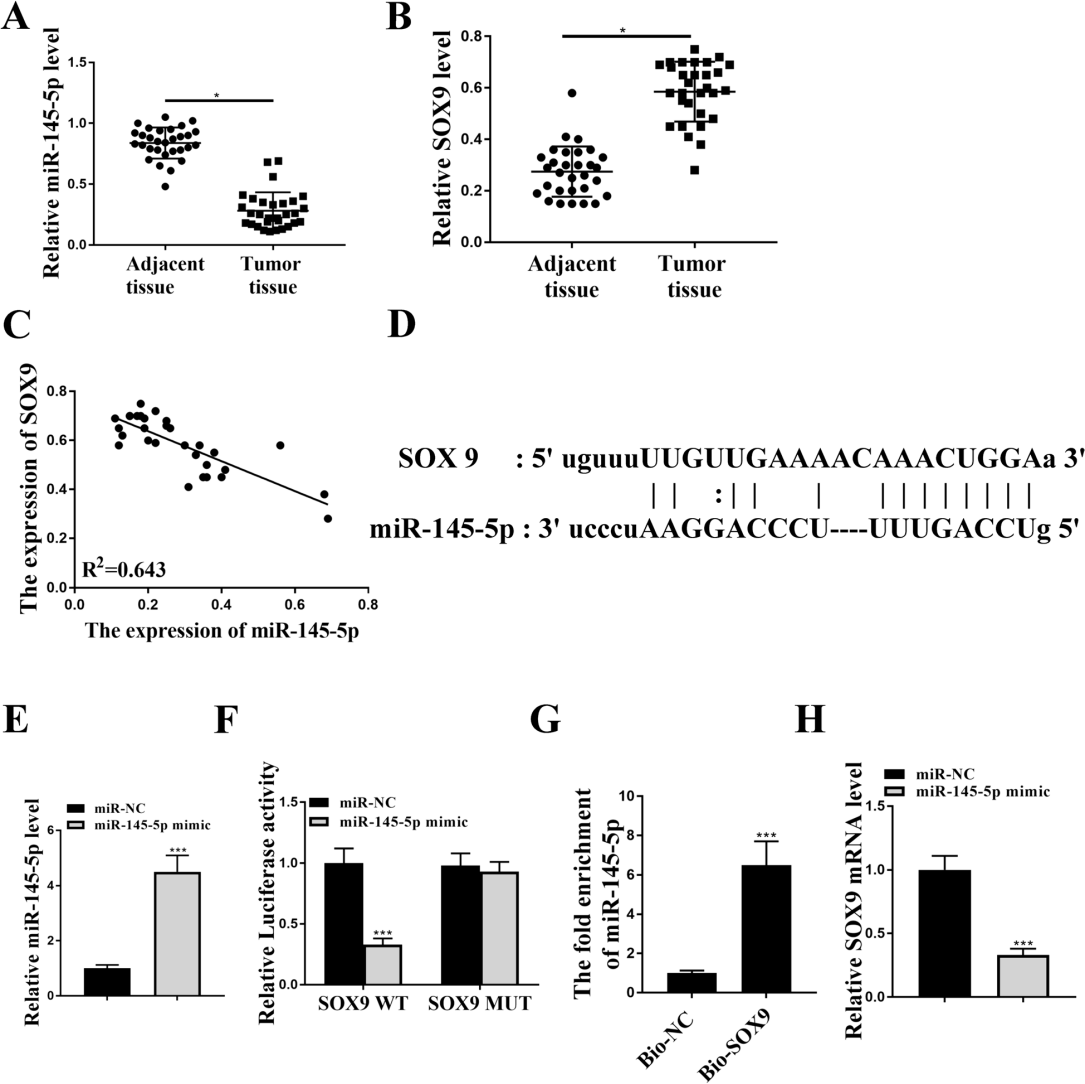
The binding relationship between miR-145-5p and SOX9. **A** and **B** QRT-PCR analysis of miR-145-5p (**A**) and SOX9 (**B**) in NSCLC tissues. **C** Correlation analysis between SOX9 and miR-145-5p expression levels in NSCLC tissues. **D** The binding region between miR-145-5p and SOX9. **E** QRT-PCR detection of miR-145-5p in H1299 cells after miR mimics transfection. **F** Luciferase reporter assay. **G** RNA pull-down assay using biotin-labelled probe. **H** qRT-PCR analysis of SOX9 in H1299 cells after miR mimics transfection. * *p* < 0.05, *** *p* < 0.001

It has been reported that miR-145-5p was a upstream miRNA of SOX9 [14]. To verify this, we assessed their expression in NSCLC tissues. qRT-PCR analysis showed that the expression levels of miR-145-5p were reduced, while the expression levels of SOX9 were increased in NSCLC tissues (Fig. 4A and B). As shown in Fig. 4C, the expression of miR-145-5p and SOX9 in NSCLC tissues were negatively correlated. The putative binding region between them was illustrated in Fig. 4D. Then we introduced miR mimics into A549 cells, and the expression of miR-145-5p was notably enhanced by mimic transfection (Fig. 4E). MiR-145-5p mimics significantly reduced the luciferase activity of SOX9 WT vector in H1299 cells, but had no impact on SOX9 MUT (Fig. 4F). Moreover, RNA pull-down assay results showed that miR-145-5p could bind to SOX9 (Fig. 4G). Meanwhile, overexpression of miR-145-5p notably reduced the expression levels of SOX9 in A549 cells (Fig. 4H). These findings confirmed the regulatory relationship of the miR-145-5p/SOX9 axis.

### Inhibition of SOX9 could reversed the promoted role of Exo-miR-145-5p inhibitor in vitro and in vivo

**Fig. 5:**
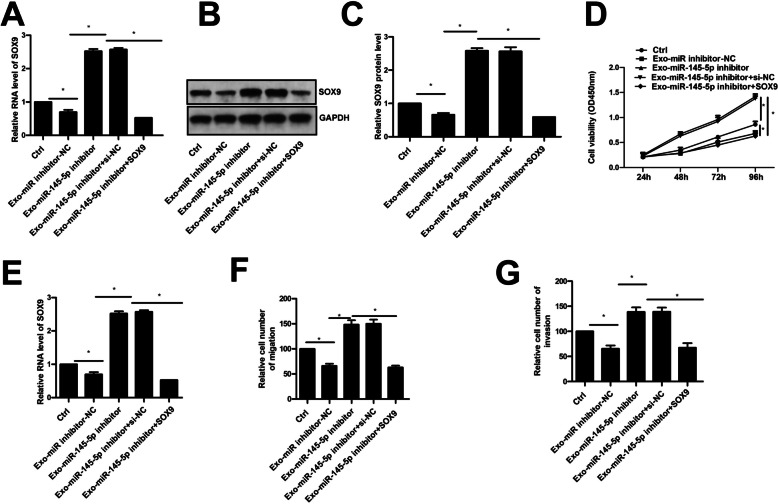
The role of SOX9 in hBMSCs-Exo miR-145-5p regulated NSCLC progression in vitro. The hBMSCs-Exo miR-145-5p mimic could upregulated the mRNA (**A**) and the protein (**B**, **C**) level of SOX9, which could be suppressed by si-SOX9. Inhibition of SOX9 could significantly reversed the suppression of NSCLC progression by hBMSCs-Exo miR-145-5p mimic, which was confirm by (**D**) CCK-8 assay, (**E**) EdU staining assay. scale bar = 40 μm, transwell assay for (**F**) cell migration and (**G**) invasion. * *p* < 0.05

**Fig. 6:**
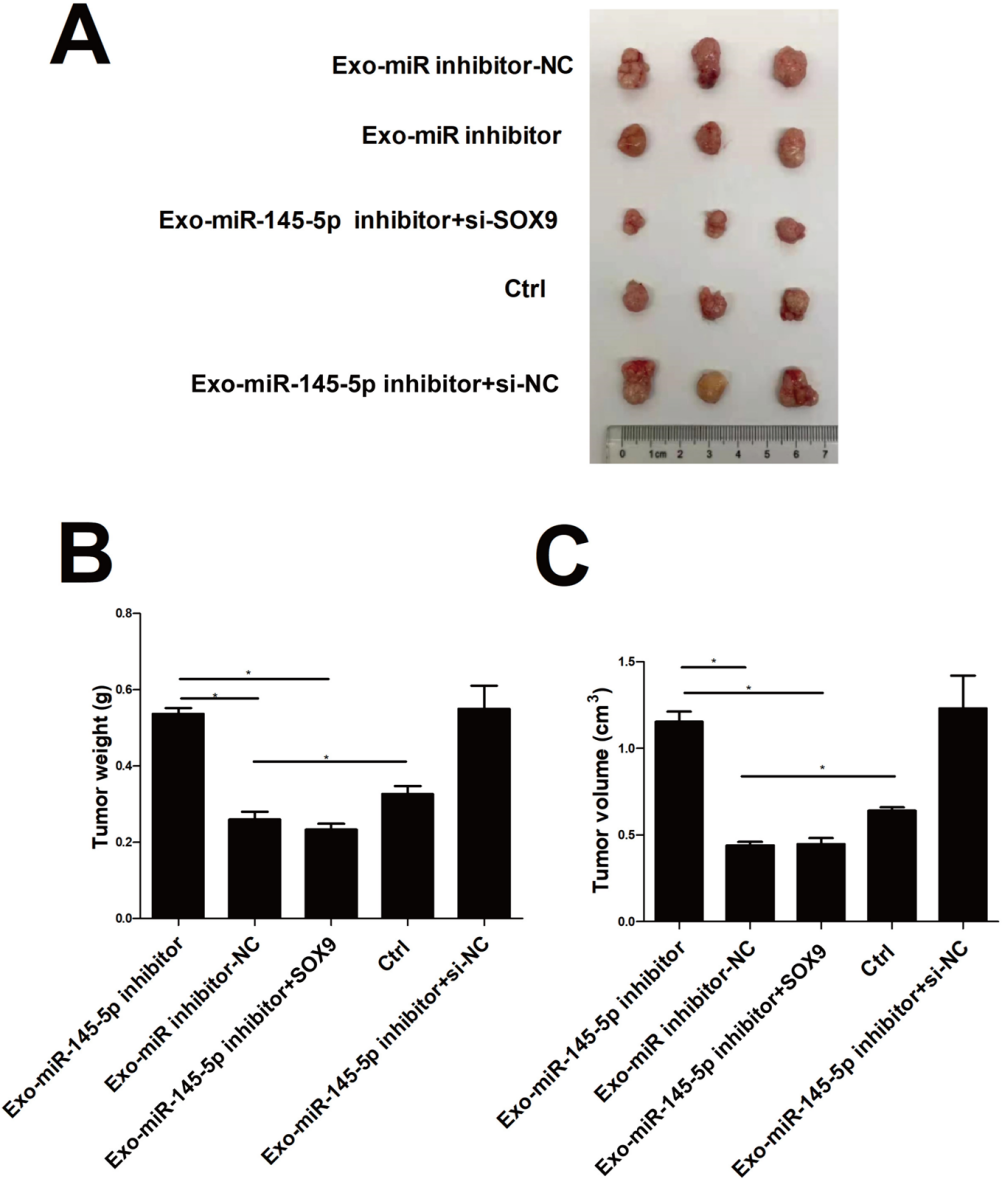
The role of SOX9 in hBMSCs-Exo with increased expression levels of miR-145-5p in NSCLC progression in vivo. **A** Tumor images. **B** Tumor volume. **C** Tumor weight. * *p* < 0.05, ** *p* < 0.01

Inhibition of miR-145-5p could up-regulated the mRNA and protein level of SOX9 in A549 cell, which was suppressed by si-SOX9 (Fig. 5A-C). Furthermore, inhibition of hBMSCs-Exo miR-145-5p significantly promoted cell proliferation, migration, and invasion of A549 cells, which could be reversed significantly by inhibition of SOX9 (Fig. 5D-G). Finally, to confirm our findings in vitro, we established an in vivo model. As shown in Fig. 6A-C, hBMSCs-Exo with miR-145-5p inhibitor promoted tumor volume and weight, which was also significantly reversed by inhibition of SOX9. These results indicated that hBMSCs-Exo could be involved in NSCLC progression via miR-145-5p/SOX9 axis.

## Discussion

Given the epidemiological significance of NSCLC as a leading cause of cancer-related death, there is a growing interest in investigating the specific mechanisms underlying this disease and exploring novel therapeutic strategies [19, 20]. In the present study, we demonstrated that hBMSCs-derived Exos could attenuate the malignant phenotypes of NSCLC both in vitro and in vivo. Furthermore, our findings clearly indicate that the inhibition of hBMSCs-derived exosomes on NSCLC progression is attributed to the enrichment of miR-145-5p in these exosomes. Additionally, there is a potential association between SOX9 and the impact of hBMSCs-derived exosomes on NSCLC progression. Interestingly, our data confirmed the tumor suppressive role of miR-145-5p in NSCLC progression, and also provided a novel therapeutic strategy for this disease.

Previous studies have found that miR-145-5p expression was significantly downregulated in NSCLC. Our results further confirmed this finding. In addition, we elucidated the delivery of miR-145-5p from hBMSCs-derived Exos. The exosome is small in size and can penetrate the biological membrane. It can be used as an ideal carrier for transporting miR-145-5p, and can protect the stability of its internal miR-145-5p to prevent degradation. Without overexpressing this miRNA, hBMSCs-derived Exos could also inhibit NSCLC progression. Except for its role in NSCLC, the impact of miR-145-5p has also been studied in other human cancers. For example, miR-145-5p suppresses the proliferative rate, the metastasis and EMT of colorectal cancer cells by negatively regulating CDCA3 [21]. MiR-145-5p can inhibit tumor growth, as well as distal metastasis in breast cancer by targeting SOX2 [22]. MiR-145-5p can also suppress the proliferative, migratory and invasive rates of cervical carcinoma cells through targeting KLF5 [23]. In addition, miR-145-5p was reported to attenuate paclitaxel resistance and prevent the progression of drug-resistant breast cancer cell lines, suggesting that it may be a novel therapeutic target in breast cancer [24]. Considering the ability of hBMSCs-derived exosomes containing miR-145-5p to mitigate NSCLC progression, it raises the question of whether a similar mechanism might also be observed in other human cancers involving miR-145-5p. These potential phenomenon and mechanism should be further investigated in future studies. In the context of NSCLC, the next challenge lies in efficiently delivering hBMSCs-derived exosomes to the tumor site within the body. We firmly believe that our findings will make a significant contribution towards the development of novel and effective therapeutic regimens for the treatment of NSCLC.

Increasing evidence has demonstrated that miRNAs exert their crucial functions in eukaryotic cells through binding to the 3’-UTR of downstream genes, followed by the inhibition in disease progression [25, 26]. In our study, we found that SOX9 was a target of miR-145-5p, and their binding relationship was verified. Furthermore, the observed alterations in SOX9 expression in NSCLC cells after miR mimics transfection in vitro, as well as in the in vivo model following the injection of hBMSCs-derived exosomes with upregulated miR-145-5p, collectively indicate that SOX9 is intricately involved in the regulatory effects of exosomal miR-145-5p on NSCLC progression. Previous studies also demonstrated that SOX9 was highly expressed in NSCLC [27, 28]. Furthermore, the inhibition of SOX9 could reversed significantly of the regulation of NSCLC by hBMSCs-derived exosomes with miR-145-5p in vitro and in vivo. Previous studies reported that miR-145-5p could target several genes, and the relevant regulatory mechanisms in human disease were also investigated. For example, miR-145-5p attenuates rheumatoid arthritis by inhibiting the Wnt1/beta-catenin signaling pathway [29]. MiR-145-5p reduces the proliferative and migratory rates of hepatocellular carcinoma cells by targeting KLF5 [30]. In addition, TLR4, TAGLN2, RHBDD1, and TGFβR2 were also identified as the downstream genes of miR-145-5p [31–34]. Our future investigations will focus on elucidating whether these known targets mediate the role of hBMSCs-derived exosomes in NSCLC progression.

In conclusion, our findings demonstrate that exosomal miR-145-5p derived from hBMSCs-derived exosomes may play a pivotal role in preventing NSCLC development by regulating SOX9. This discovery offers a promising avenue for the development of a novel therapy for this disease.

### Supplementary Information

Supplementary Material 1.

Supplementary Material 2.

Supplementary Material 3.

Supplementary Material 4.

Supplementary Material 5.

Supplementary Material 6.

## Data Availability

No datasets were generated or analysed during the current study.
