# Supplementary figures and images for: Bone marrow mesenchymal stem cells-derived exosomal miR-145-5p reduced non-small cell lung cancer cell progression by targeting SOX9
Source: BMC Cancer. 2024 Jul 22;24:883. doi: 10.1186/s12885-024-12523-z (PMC11265358; doi:10.1186/s12885-024-12523-z)

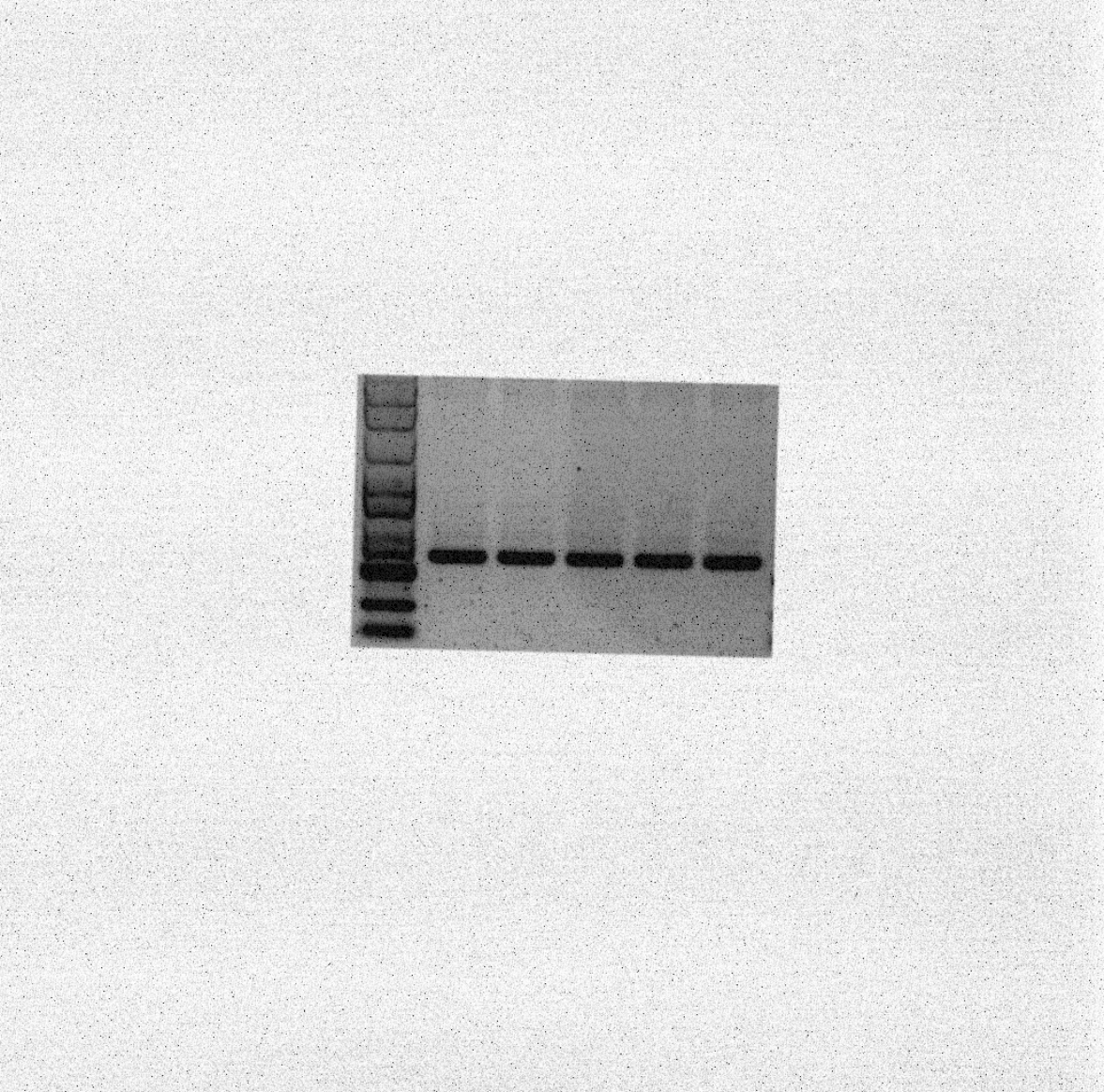

Supplement: Supplementary file 1 — Supplementary Material 1. [file 12885_2024_12523_MOESM1_ESM.tif]

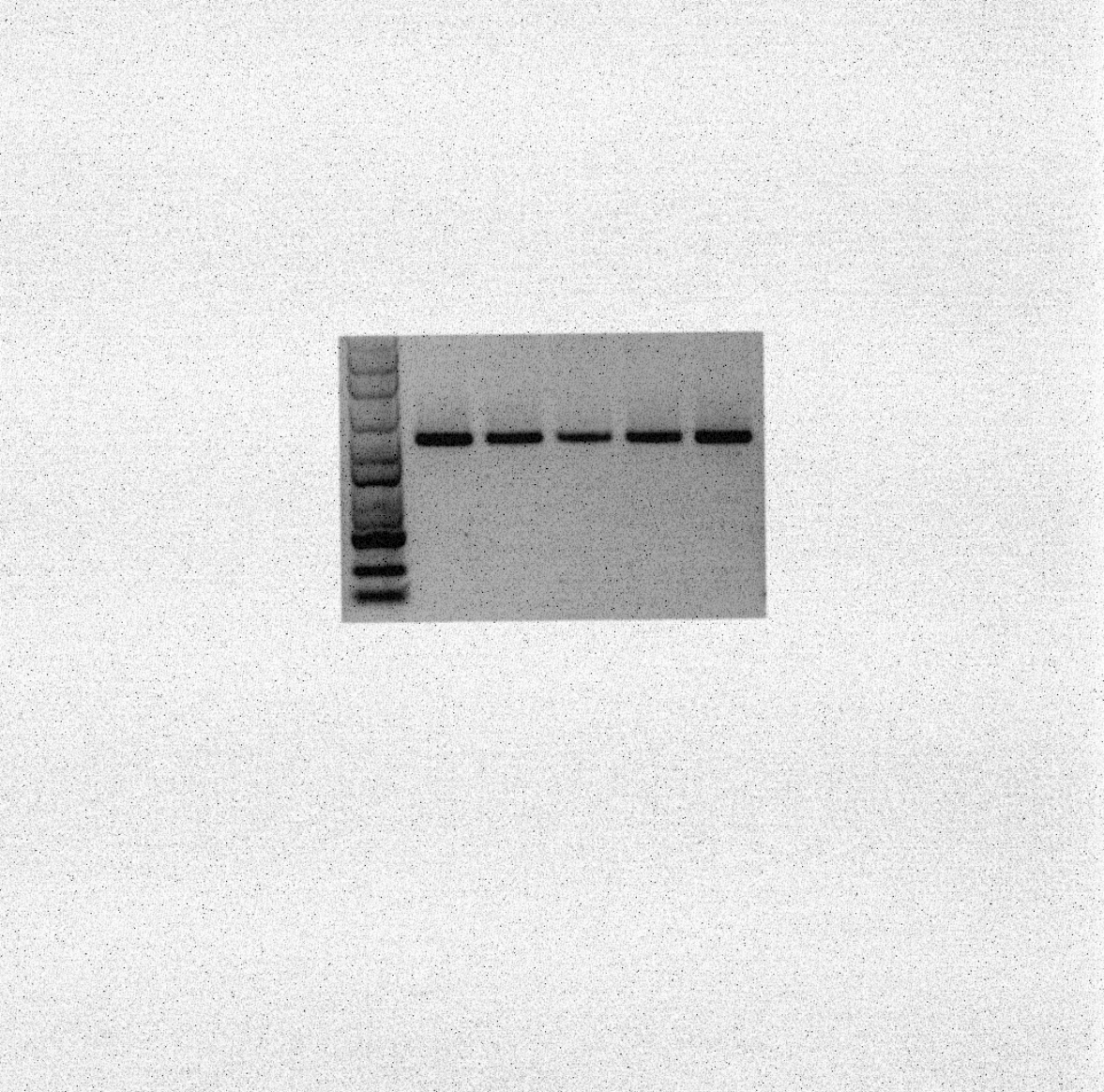

Supplement: Supplementary file 2 — Supplementary Material 2. [file 12885_2024_12523_MOESM2_ESM.tif]

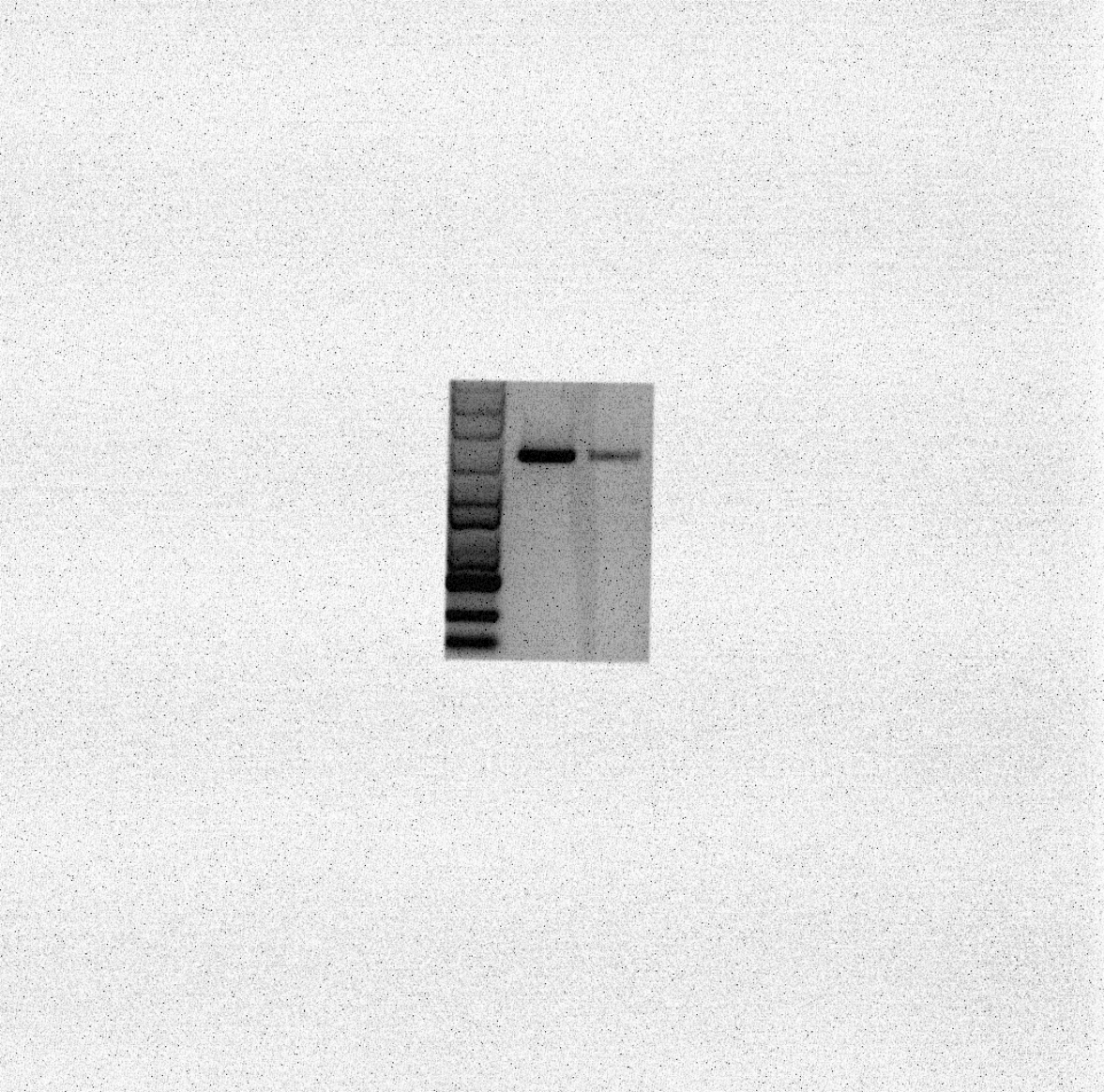

Supplement: Supplementary file 3 — Supplementary Material 3. [file 12885_2024_12523_MOESM3_ESM.tif]

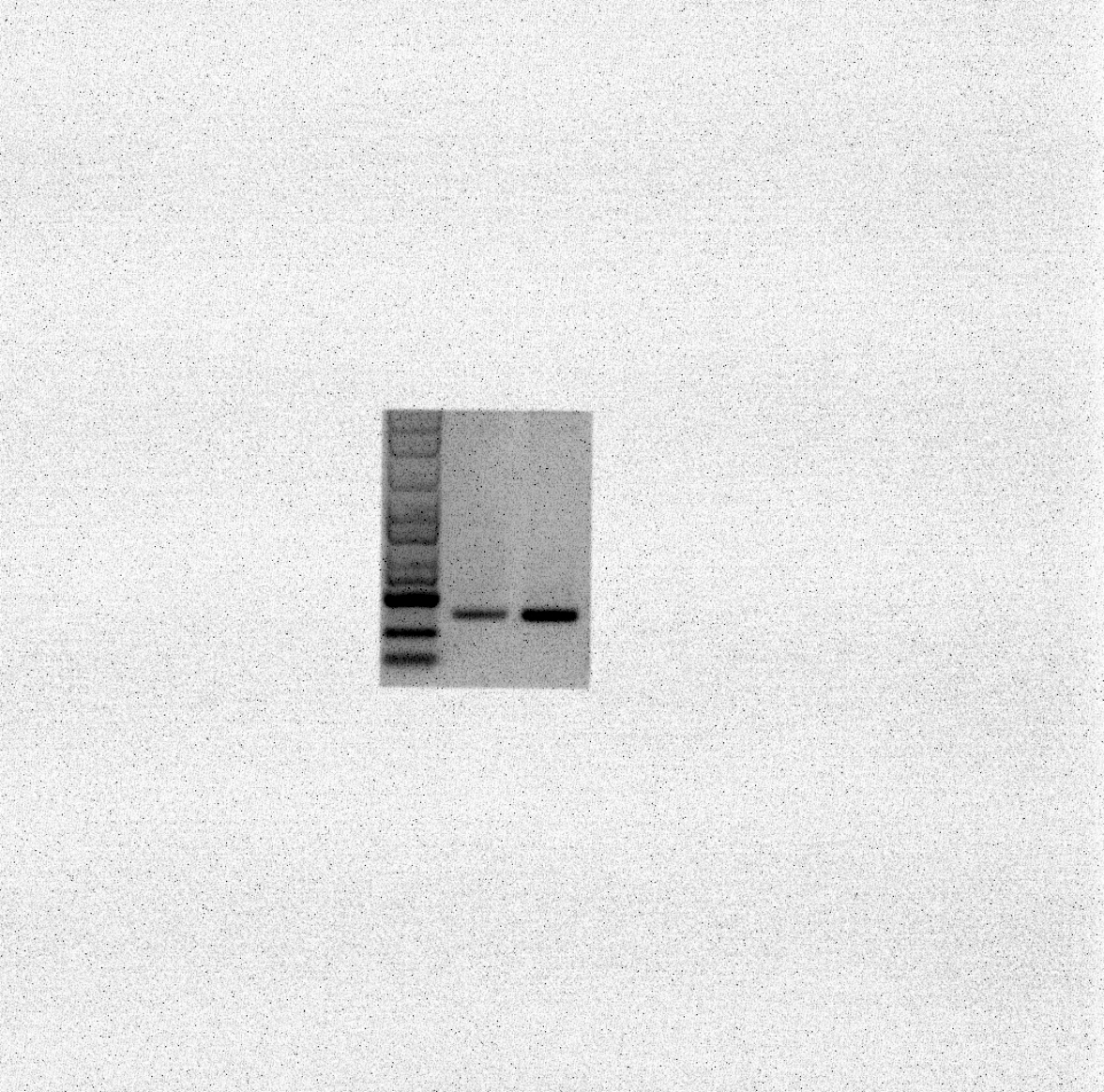

Supplement: Supplementary file 4 — Supplementary Material 4. [file 12885_2024_12523_MOESM4_ESM.tif]

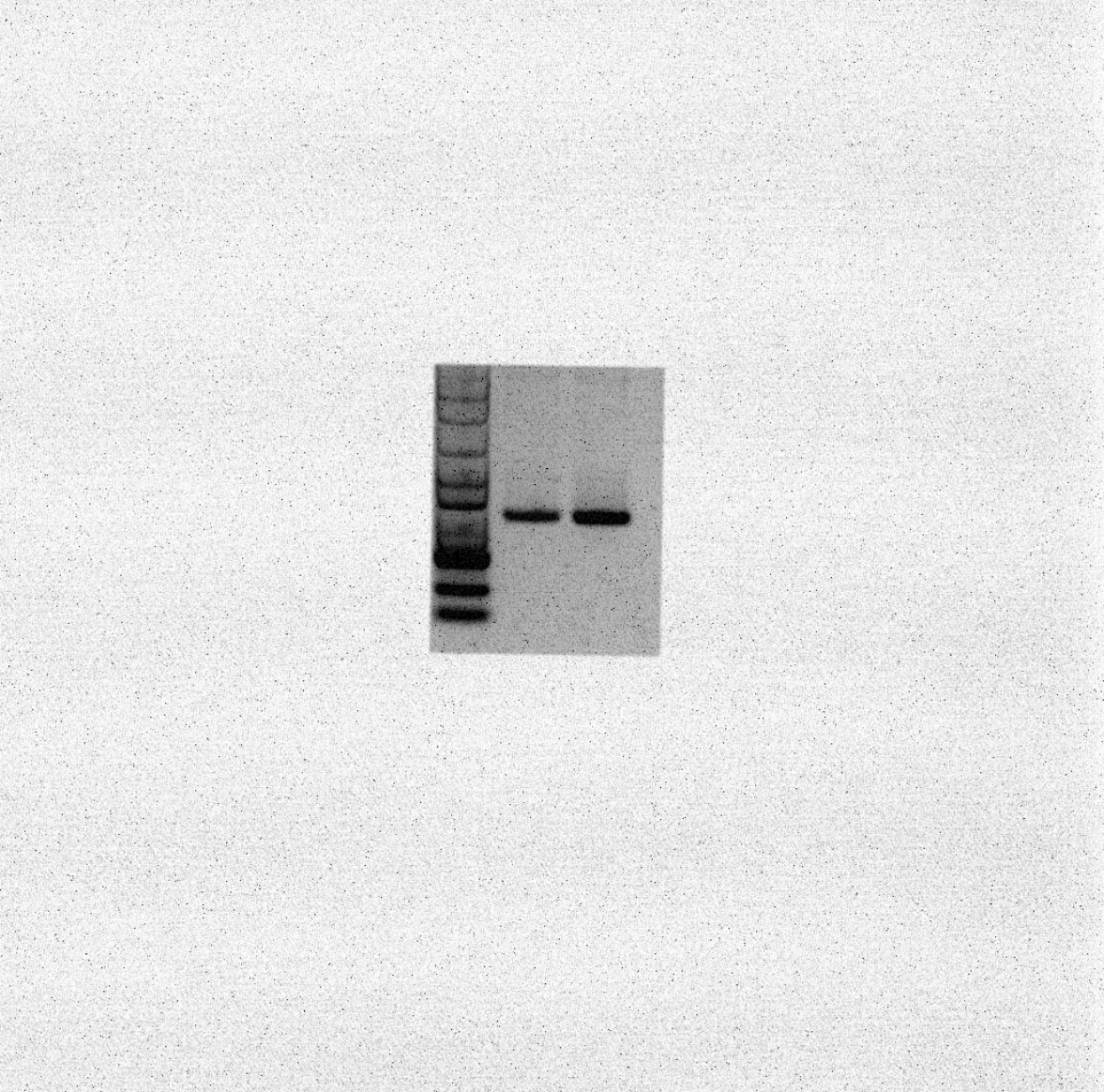

Supplement: Supplementary file 5 — Supplementary Material 5. [file 12885_2024_12523_MOESM5_ESM.tif]

GAPGH


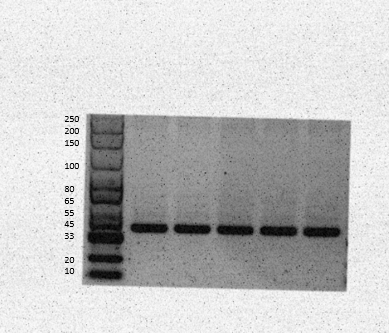


SOX9


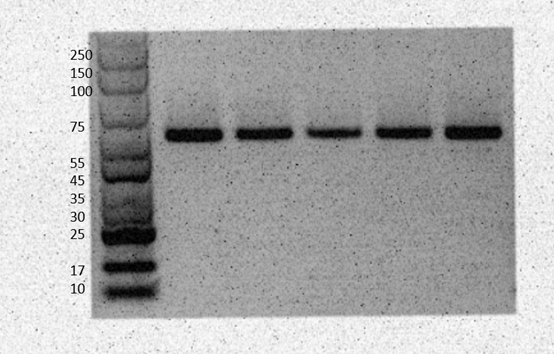


CLANEXIN


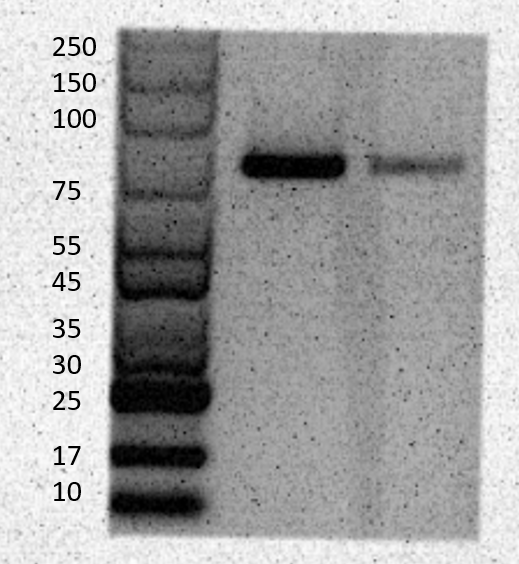


CD63


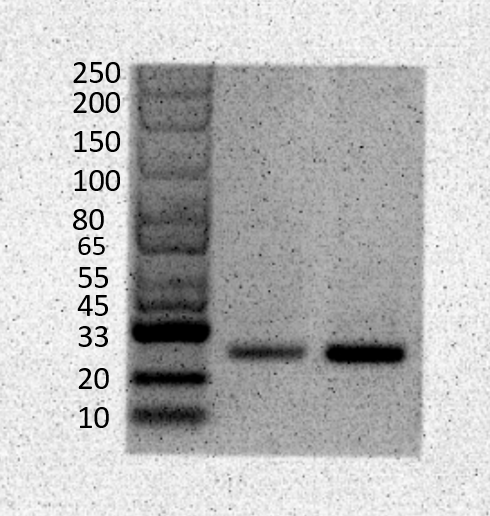


TGC101


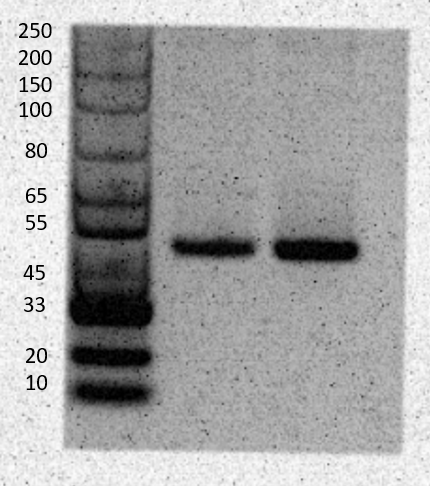

Supplement: Supplementary file 6 — Supplementary Material 6. [file 12885_2024_12523_MOESM6_ESM.docx]
